# E-Cigarette Use by Smoking Status in Estonia, 2012–2018

**DOI:** 10.3390/ijerph17020519

**Published:** 2020-01-14

**Authors:** Rainer Reile, Kersti Pärna

**Affiliations:** 1 Department of Epidemiology and Biostatistics, National Institute for Health Development, 11619 Tallinn, Estonia; 2 Institute of Family Medicine and Public Health, University of Tartu, 50411 Tartu, Estonia

**Keywords:** e-cigarettes, tobacco policy, smoking, Estonia

## Abstract

**Background:** In the context of declining smoking rates in Estonia, this study aims to analyze the recent trends in e-cigarette use and its associations with smoking status and sociodemographic factors. **Methods:** Nationally representative data from biennial cross-sectional health surveys in 2012–2018 (n = 9988) were used to describe the prevalence of smoking and e-cigarette use by smoking status in Estonia. Multivariate logistic regression analysis was used to describe the sociodemographic patterns of e-cigarette use in three subgroups: the general population, smokers, and ex-smokers. **Results:** The prevalence of current smoking decreased from 45.4% in 2012 to 31.5% in 2018 among men and from 26.6% to 20.0% among women. At the same time, e-cigarette use in the general population had increased to 3.7% among men and to 1.2% among women. The increase in the prevalence of e-cigarette use was statistically significant among men in the general population, smokers, and ex-smokers, but non-significant among women. In addition to period effects, e-cigarette use was patterned by age, gender, and education. **Conclusion:** In 2002–2018, the e-cigarette use had increased but smoking had decreased in Estonia. A timely and targeted tobacco policy may alleviate the harm of e-cigarette use from the public health perspective.

## 1. Introduction

Estonian tobacco legislation has witnessed several significant changes over the past decades that have had a positive effect on overall smoking rates. Since the implementation of the Tobacco Act in 2001 and its later revisions [1], the prevalence of current smoking among 16–64 year old adults had decreased during 2000–2016 from 53.5% to 39.3% among men and from 28.3% to 22.3% among women [2]. More recent data from 2018 [3] suggests a further decline, now with 31.5% of men and 20.0% of women being current smokers—figures close to WHO’s projected estimates for 2025 [4].

Electronic nicotine delivery systems (ENDS), commonly known as e-cigarettes, have become increasingly popular in recent years [5]. As they do not involve inhaling and exhaling the fumes of burning plant material, their use is not considered de jure as smoking and are hence not included when calculating prevalence estimates for smoking [3].

While there is lack of consensus on the long-term harms or benefits of e-cigarettes in terms of smoking uptake or cessation, the majority of e-cigarette users are either current or ex-smokers [6,7,8]. Available evidence suggests that e-cigarettes serve as a possible gateway to cigarette smoking as demonstrated by a recent study [9] where non-smoking adolescent e-cigarette users had higher odds of subsequent smoking. Moreover, the concurrent use of e-cigarettes does not necessarily lead to higher quit rates. On the contrary, an earlier meta-analysis found that the odds of quitting cigarettes were 28% lower in those who used e-cigarettes compared with those who did not use e-cigarettes [10]. Given the accumulating evidence on the health risks of e-cigarettes [11,12], the increasing popularity of e-cigarettes presents a significant public health concern.

The present study is motivated by a hypothesis that the previously described decline in smoking prevalence could—at least partly—be explained by the increasing prevalence of e-cigarettes. Therefore, we will describe the recent trends in e-cigarette use among the adult population of Estonia and analyze its associations with smoking status and sociodemographic factors.

## 2. Methods

Data from nationally representative cross-sectional postal surveys of Health Behavior among Estonian Adult Population in 2012–2018 were used for this analysis. All surveys were approved by the Tallinn Medical Research Ethics Committee. The detailed description of survey methodology is available elsewhere [3]. This study included 9988 adults aged 16–64 years (4967 men and 5021 women) who had provided data on their a) smoking status, and b) e-cigarette use (daily or several times weekly vs. seldom or never). The prevalence of e-cigarette use, with 95% confidence intervals (95% CI) among the general population, smokers, and ex-smokers, was calculated separately for men and women with a Z-test used for a comparison of proportions. Multivariate logistic regression analysis was performed to compare the potential socio-demographic variations in e-cigarette users vs. non-users among: (a) the general population, (b) smokers, and (c) ex-smokers. Independent variables included sex, age, education, study year, and smoking status (smoker or non-smoker). The latter was used as a predictor variable for analysis on the general population and as a stratifying variable for other models. The results are presented as odds ratios (OR) with 95% confidence intervals (CI). This study used population weights to compensate for the response bias. Statistical analyses were conducted using SPSS Statistics for Windows, version 25.0 (IBM Corp., Armonk, NY, USA, 2017).

## 3. Results

During 2012–2018, the prevalence of current smoking among 16–64-year-old men declined from 45.4% (95% CI 42.8–48.0) to 31.5% (95% CI 28.9–34.1). Among women, the change was slightly smaller, with 20.0% (95% CI 17.9–22.3) being smokers in 2018 compared to 26.6% (95% CI 24.4–28.9) in 2012. The decline in smoking rates was statistically significant for both genders.

**Figure 1:**
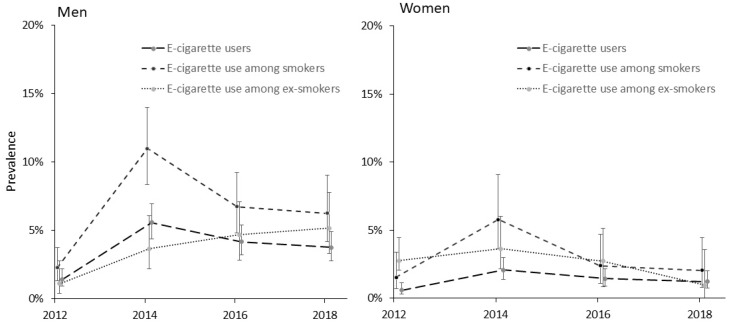
Prevalence of e-cigarette use in Estonia, 2012–2018.

The same period saw a statistically significant increase in e-cigarette use (Figure 1). Among men, the overall prevalence of e-cigarette use increased from 1.4% (95% CI 0.9–2.2) in 2012 to 3.7% (95% CI 2.8–4.9) in 2018. Among women, the e-cigarette use increased from 0.6% (95% CI 0.3–1.1) in 2012 to 1.2% (95% CI 0.7–2.0) in 2018, but the change was statistically not significant.

Among the smoking population, a statistically significant increase in e-cigarette use during 2012–2018 was found only for men. In 2018, e-cigarettes were regularly used by 6.3% (95% CI 4.2–9.0) of smoking men compared to 2.3% (95% CI 1.3–3.7) in 2012. Among smoking women, e-cigarette use was lower (2.0% (95% CI 0.8–4.4) in 2018), and the respective increase from 2012 levels (1.5%; 95% CI 0.7–3.4) was very subtle. Similarly, an increase in e-cigarette use over the study period was observed for non-smoking men. In 2018, 5.2% (95% CI 3.3–7.8) of non-smoking men used e-cigarettes regularly compared to just 1.1% (95% CI 0.4–2.8) in 2012. For non-smoking women, the respective change was from 0.8% (95% CI 0.2–2.3) in 2012 to 2.7% (95% CI 1.3–5.2) in 2018.

The sociodemographic and temporal variations in e-cigarette use are given in Table 1. Among the general population, higher odds for using e-cigarettes were found for men compared to women (OR 2.3; 95% CI 1.7–3.1) and at younger ages. Although the educational differences were statistically non-significant in the adjusted analysis, differences in e-cigarette use were found for study year and smoking status variables. While the period effects follow the previously described prevalence trends, smokers had 3.6 times higher odds for being an e-cigarette user than non-smokers.

Among smokers, e-cigarette use was significantly higher among men compared to women (OR 2.4; 95% CI 1.6–3.5) and in the youngest age group compared to 45–64-year-olds (OR 2.3; 95% CI 1.1–4.7). Compared to 2012, the relative odds for e-cigarette use among smokers were highest in 2014 (OR 5.0; 95% CI 3.0–8.4).

Among ex-smokers, higher relative odds for e-cigarette use were found in younger age groups (*vs.* 45–64-year old) and among those with secondary (vs. tertiary) education, but differences in e-cigarette use across the genders were non-significant. Compared to 2012, the use of e-cigarettes was higher in later study years.

## 4. Discussion

The present study analyzed the trends of e-cigarette use in Estonia where smoking rates among the adult population have substantially decreased in recent years. The e-cigarette use in the general population had increased by 2.3% among men and by 0.6% among women during 2012–2018. Although the overall change was only statistically significant for men, a larger increase was seen among ex-smokers. While these results indicate that e-cigarettes are becoming increasingly popular, their use is patterned by sociodemographic variables.

Before discussing these findings, some potential considerations regarding the data need to be addressed. Firstly, the study is based on repeated cross-sectional data that do not allow establishing causal pathways between e-cigarette use and smoking status. Also, the selection and operationalization of indicators may potentially have affected the results. This is especially relevant for variables on e-cigarette use and smoking status. As the first was initially a frequency variable measured on a 5-point Likert scale, we tested different combinations of these options (e.g., daily/almost daily vs. daily/almost daily or a few times a week) beforehand to ensure the robustness of the results. Regarding the smoking status, e-cigarette use was very low among respondents who had never smoked. Respective analysis of this subgroup was therefore not performed. The methodological consistency of the survey data could be considered as a strength of the study, but the overall response rates (ranging from 51% in 2018 to 62% in 2012) have been declining across the survey years. We used population weighting to assure data representativeness and reduce the potential non-response bias. Although the latter cannot be fully avoided, a previous study using the same survey data demonstrated that response bias had a minimal effect on prevalence indicators [13].

The results indicate that e-cigarette use has increased significantly since 2012 among men irrespective of smoking status. Although the same period saw a substantial decline in smoking prevalence (e.g., 13.9% among men), the concurrent but opposite trends of smoking and e-cigarette use in Estonia cannot be interpreted causally in our data. However, the decline in smoking prevalence surpassed the increase in e-cigarette use in absolute terms. Despite this, the relative change in e-cigarette use—nearly a three-fold increase among smoking men and almost a five-fold increase in non-smokers—is noteworthy.

The prevalence is also slightly higher than in other comparable studies. For example, a study from the United States [14] found that only 0.9% of adults were regular e-cigarette users in 2014, whereas e-cigarettes were regularly used by 5.8% of Estonian men and 2.1% of women in 2014. More recently, Eurobarometer 458 study [15] reported that the highest prevalence (5.0%) of current e-cigarette users in Europe is in the United Kingdom. In this context, the comparable rates of e-cigarette use in Estonia are a cause for concern.

The use of e-cigarettes peaked in 2014 and has since then been declining (except for male ex-smokers). This is explained by deregulating the sale of e-cigarettes and refill liquids in 2013 [16]. These were, since 2007, marketed as medicinal products, greatly constraining their sales. In 2013, all refill liquids with a nicotine content below 2 mg/mL or liquids with a nicotine content up to 4 mg/mL without a specific medical prescription were allowed for retail distribution. The wide availability, media coverage, and also advertising [16] resulted in a sharp increase in e-cigarette use, as also demonstrated by our data. The 2015 revision of Tobacco Act [1] defined e-cigarettes as “products related to tobacco products” and ensued several restrictions to the sale, marketing, and the use of e-cigarettes and refill liquids. Since 2018, the use of e-cigarettes in public spaces is also prohibited, similar to smoking, in order to further restrict their use.

In addition to the previously described period effects, the use of e-cigarettes was patterned by gender (in the general population and smokers), age, and education (in the general population and ex-smokers). Although previous studies have shown mixed findings regarding gender differences in e-cigarette use [5,17], we found a considerably higher e-cigarette use among men. Moreover, e-cigarette use was more prevalent among smokers and ex-smokers compared to the general population. This supports the earlier evidence [6] that many smokers use e-cigarettes concurrently with cigarette smoking. Also, in accordance with earlier studies [18,19], the youngest age group had the highest odds for using e-cigarettes among the general population, but also among smokers and ex-smokers. This may entail a considerable challenge for public health, as recent meta-analysis [20] demonstrated a greater risk for subsequent cigarette smoking among adolescents who use e-cigarettes. E-cigarettes serve as a possible gateway to cigarette smoking [5,21]. The age-difference was even larger for ex-smokers, indicating a potentially higher substitution of conventional smoking with e-cigarettes in younger ages despite the evidence that use of e-cigarettes is not associated with reduced overall quit rates compared to exclusive smoking [22]. Unfortunately, our cross-sectional data does not include data on the timing of smoking cessation or on the uptake of e-cigarette use, and this claim cannot be studied in a more detailed manner. Although higher education and income have been associated with higher e-cigarette use [5], the inverse educational effects found in our data for the ex-smoking population is explained by the interaction’s effects on age—the e-cigarette use is highest among the youngest age group who haven’t completed their education yet.

## 5. Conclusion

The use of e-cigarettes has increased substantially during 2012–2018 among adult men in Estonia. Despite the opposite trend found for smoking prevalence, it is not certain that the increasing popularity of e-cigarettes explains the decrease in smoking rates in Estonia. However, the e-cigarette use varies greatly by smoking status and sociodemographic background. The higher e-cigarette use among young adults calls for a timely and targeted tobacco policy to alleviate the harm of e-cigarette use from the public health perspective.

## Figures and Tables

**Table 1:** Mutually adjusted OR with 95% confidence intervals (CI) by smoking status and e-cigarette use.

Characteristic	General Population E-Cigarette Use vs. Non-Use		Smoking Population E-Cigarette Use vs. Non-Use		Ex-Smoking Population E-Cigarette Use vs. Non-Use	
	n	OR (95% CI) *	n	OR (95% CI) *	n	OR (95% CI) *
**Sex**						
Women	5828	1	1365	1	1369	1
Men	4084	**2.30 (1.72–3.08)**	1606	**2.37 (1.60–3.50)**	1230	1.52 (0.96–2.42)
**Age**						
45–64	3308	1	930	1	951	1
35–44	3103	1.22 (0.83–1.79)	915	1.09 (0.69–1.71)	831	1.83 (0.86–3.89)
20–34	2939	**1.89 (1.33–2.69)**	981	1.26 (0.82–1.94)	739	**4.77 (2.43–9.38)**
16–19	562	**2.90 (1.69–4.99)**	145	**2.31 (1.14–4.69)**	79	**9.94 (3.68–26.84)**
**Education**						
Tertiary	1782	1	271	1	476	1
Secondary	7081	1.32 (0.85–2.04)	2227	0.63 (0.36–1.11)	1905	**2.38 (1.17–4.85)**
Primary	1049	1.36 (0.78–2.38)	473	0.70 (0.35–1.37)	218	1.62 (0.57–4.56)
**Study year**						
2012	2623	1	917	1	609	1
2014	2377	**4.66 (2.98–7.30)**	712	**4.99 (2.98–8.38)**	661	**3.67 (1.46–9.23)**
2016	2529	**3.55 (2.22–5.67)**	744	**2.46 (1.40–4.35)**	670	**5.67 (2.28–14.06)**
2018	2383	**3.51 (2.17–5.70)**	598	**2.25 (1.22–4.12)**	659	**5.97 (2.40–14.88)**
**Smoking status**						
Smoker	2971	**3.55 (2.70-4.67)**	na		na	
Non-smoker	6869	1	na		na	

* Statistically significant (*p* < 0.05) associations are given in bold.
